# Economic Burden Conferred by Population-Level Cancer Screening on Resource-Limited Communities: Lessons From the ESECC Trial

**DOI:** 10.3389/fonc.2022.849368

**Published:** 2022-03-21

**Authors:** Fuxiao Li, Yanjun Hu, Chuanhai Guo, Liang Lei, Fenglei Li, Mengfei Liu, Zhen Liu, Yaqi Pan, Fangfang Liu, Ying Liu, Zhe Hu, Huanyu Chen, Zhonghu He, Yang Ke

**Affiliations:** ^1^ Key Laboratory of Carcinogenesis and Translational Research (Ministry of Education/Beijing), Laboratory of Genetics, Peking University Cancer Hospital & Institute, Beijing, China; ^2^ Shenzhen Institute of Advanced Technology, Chinese Academy of Sciences, Shenzhen, China; ^3^ Healthcare Security Administration of Hua County, Anyang, China; ^4^ Hua County People’s Hospital, Anyang, China

**Keywords:** upper gastrointestinal cancer screening, treatment costs, catastrophic medical expenditure, health insurance sectors, randomized controlled trials

## Abstract

**Objectives:**

Upper gastrointestinal (G.I.) cancer screening has been conducted in China for decades. However, the economic burden for treatment “intensively” occurred in advance due to screening in resource-limited communities remain unclear.

**Methods:**

We compared the treatment costs for upper G.I. cancers from the screening and control arms of a population-based randomized trial in a high-risk area for esophageal cancer (EC) in China based on claims data from the health insurance system in the local area which included whole population coverage.

**Results:**

The average out-of-pocket cost per treatment of EC in the screening arm was lower than that in the control arm ($5,972 *vs.* $7,557). This difference was a consequence of down-staging from screening which resulted in lower cost therapy for earlier stage cancers. Moreover, this result is similar for cardial and non-cardial gastric cancer in the two study arms ($7,933 *vs.* $10,605). However, three times as many (103 *vs.* 36) families in the screening arm suffered catastrophic health expenditure for all cancer types. The overall treatment cost for all EC patients in the screening arm ($1,045,119) was 2.44 times that in the control arm ($428,292), and the ratio for cardial and non-cardial gastric cancer was 1.12 ($393,261 *vs.* $351,557).

**Conclusion:**

Cancer treatment secondary to screening may triple the likelihood of catastrophic patient medical expenditure, and sharply increase the economic pressure on the local community, particularly for cancer types which are of high prevalence. Financial support for patients and the health insurance system should be taken into consideration when planning budgets for cancer screening programs in communities which are resource-limited.

## Introduction

Cancer is the second leading cause of death globally and accounted for an estimated 9.6 million deaths in 2018 (1). Etiologic factors for cancers are incompletely understood, and extensive resources have been invested in cancer screening (2). Currently, economic evaluation is widely used to guide the formulation of cancer-screening strategies (3). However, in screening programs without therapeutic intervention, government budgets are mainly focused on the screening phase, and high expenses for treatment therefore fall directly on the patients themselves and/or on local insurance sectors. This may cause unanticipated problems that cannot be well accounted for by traditional cost-effectiveness analysis. On the one hand, patients from low-income households may easily be impoverished by catastrophic health expenditures for cancer treatment, even after medical reimbursement, resulting in deterioration of quality of life (4, 5). Moreover, financial pressure may obstruct timely treatment and hamper the effectiveness of screening programs in the real world (6). On the other hand, significant increases in treatment costs which result from cancer screening may result in a financial deficit in local medical insurance systems. Such problems are unfortunately not strongly enough emphasized in current studies of cancer screening strategies. Hence, more attention should be given to the economic burden consequent to screening intervention, especially in areas of limited resources.

Several large-scale cancer-screening programs have been carried out in China since 2000, including upper G.I. cancer screening for high-risk populations. Over 2.16 million participants had accepted endoscopic screening by 2018, but most patients with cancer detected by screening received no financial support for subsequent treatment under current screening policies (7–10). Most studies estimated treatment costs for upper G.I. cancers in order to calculate total cost-effectiveness of the screening program (8, 11–13). However, few of these studies have thoroughly investigated the economic burden of cancer treatment specifically on patient households and the local health insurance system. Lack of focused evaluation on this problem may engender unanticipated financial consequences, and lead to suboptimal screening effectiveness. Formulation of comprehensive cancer screening policies must allow for the potential impact of treatment, which entails evaluation of the economic burden brought about by screening.

In this study, we used claim records in the medical insurance system with high population coverage to extract information on treatment costs for upper G.I. cancers in an endoscopic screening trial which was randomized and controlled in a poverty-stricken county of northern China. Costs were calculated from the patients’ perspective and the societal perspective respectively, and were analyzed at both the individual level and the government level. Upper G.I. endoscopic examination could detect esophageal cancer simultaneously with cardial and non-cardial gastric cancer, and cost analysis was stratified by these two major types of cancer to distinguish the economic impact of screening for cancers of different incidence in the target population (12.45/10^5^ for cardial and non-cardial gastric cancer *vs.* 25.58/10^5^ for esophageal cancer) (14). Comparisons were conducted between patients from the screening and control arms of the study. We aimed to investigate the underappreciated financial impact brought about by cancer screening in resource-limited regions, and provide real-world evidence for design of cancer control policy.

## Methods and Materials

### Parent Study

In January 2012, we initiated the ESECC (Endoscopic Screening for EC in China) randomized controlled trial (NCT01688908) in Hua county, Henan Province, which is a region of high-risk for esophageal squamous cell carcinoma (ESCC) in northern China. The gross domestic product per capita (¥18,079 in 2017) of this region was far below the national average level (¥59,201 in 2017) (15, 16). The design of this study has been reported elsewhere (7). Briefly, 668 target villages were randomly selected and allocated into the screening arm or control arm at a ratio of 1:1 (334 villages in each arm) with 17,151 individuals in the screening arm and 16,797 in the control arm. Residents in the screening arm aged 45–69 years who had no history of cancer or endoscopic examination within five years were assigned to undergo an endoscopic examination with iodine staining. No endoscopic screening was undertaken in the control arm.

### The New Rural Cooperative Medical Scheme System

The New Rural Cooperative Medical Scheme (NCMS) system is a government-run health insurance program in rural China with population coverage of nearly 100%. NCMS claims data were recorded and uploaded in a real-time manner in health facilities where inpatients were diagnosed and treated.

### Identification and Verification of Study Subjects

Upper G.I. cancer cases from the ESECC cohort were found through endoscopic screening or clinical visits. Patients in the screening arm included screening-detected and clinically diagnosed cases, while patients in the control arm were all diagnosed clinically. Annual active follow-up and passive linkage with claims data were jointly adopted to identify clinically diagnosed cases, and the efficiency of this approach has been evaluated (14, 17). Participants identified as upper G.I. cancer cases and matched with NCMS claims records for treatment were included in this evaluation. Name, address, gender, and ID number were used to match cases in the NCMS system from January 1, 2012 to December 31, 2018. The following criteria were adopted to ensure that clinical cases were in fact diagnosed with upper G.I. cancers: 1) the diagnosis in the NCMS system was upper G.I. cancer, or a related precancerous lesion (C15, C16, D00, K22); 2) therapies recorded in the expense details were related to upper G.I. cancer treatment.

### Construction of a “Perfect Cohort” and “Standard Observation Window”

As in other community-based cluster randomized trials, enrollment for the two arms of the ESECC trial was not strictly parallel at the individual level. Participants in the screening arm were enrolled earlier and had longer follow-up time. Thus, the control arm tended to generate fewer clinically diagnosed cases within shorter follow-up periods after enrollment up to 31 December 2018; and these cases also had fewer medical records and costs collected during a shorter observation window for treatment (after the first hospitalization up to 31 December 2018). To ensure inter-group comparability, we constructed a “Perfect Cohort” which included cancer cases from both arms of the study, and captured a “Standard Observation Window” to collect treatment records and costs.

**Figure 1:**
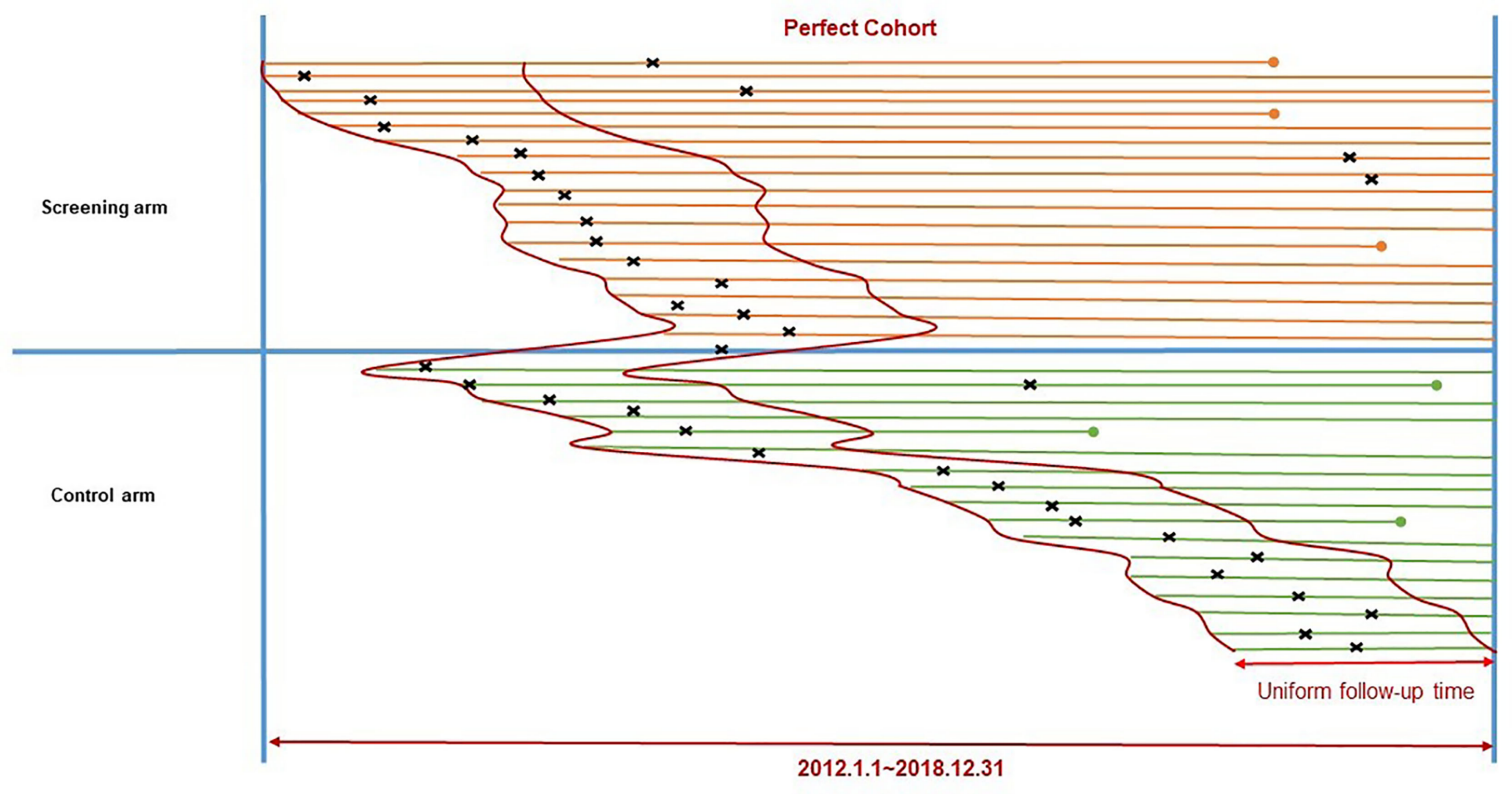
Construction of the “Perfect Cohort” to include cancer cases from the ESECC trial. Segments refer to time periods of follow-up for participants in the two study arms. The left endpoint of a segment refers to the time of enrollment, and the right endpoint refers to the end of follow-up (2018.12.31) or death (a dot). **×** The symbol “×” above each segment refers to the time of diagnosis for upper GI. cancer.

We first identified the shortest follow-up time (857 days) among all verified cancer cases and set it as the uniform follow-up time for the “Perfect Cohort”. Only those patients diagnosed within the uniform follow-up time after enrollment were included, which allowed investigation of two arms with comparable numbers of cancer cases which fell into the same follow-up time period. (**Figure 1**) A selection bias analysis was conducted which compared excluded cases and those cases which remained in the “Perfect Cohort”.

Secondly, a “Standard observation window” was needed for subjects in the “Perfect Cohort”. For each patient, the first hospitalization with a discharge diagnosis of upper G.I. cancer in a secondary (county-level) or tertiary (city-level and above) health care facility was chosen as the starting point of treatment. We explored the temporal trend of treatment costs by calculating the average treatment cost per patient under observation in each month after treatment began, as in Eq. (1) below:


(1) $$Average\;treatment\;cost\;in\;a\;given\;month\;since\;first\;hospitalization\;=\frac{\text{Total treatment cost in a given month }}{\text{number of patients under observation in this month}}\;$$

Average monthly treatment cost showed an “L-shaped” pattern, which peaked at the beginning and sharply declined to almost zero at around 12 months, and thereafter remained close to zero (**Supplementary Figure 1**). A “standard observation window” of one year was thus adopted.

### Data Collection

Each claims record contained the name of the health facility, date of admission and discharge, doctor in charge, discharge diagnosis, total expenses, reimbursed expenses, and out-of-pocket expenses. Cost details attached to the record displayed information for all medical services used during hospitalization, including the date, classification, quantity, and unit cost, together with the out-of-pocket part of the cost. Original clinical information such as cancer site, stage at diagnosis, and therapy were collected from medical records in the hospital. Socio demographic factors including age, gender, education, occupation, and yearly household income were extracted from the database of the ESECC trial.

### Cost Calculation

Two perspectives were adopted in cost calculations. From the patient perspective, we calculated total out-of-pocket costs of all cancer-related hospitalizations. From a social perspective, we calculated the treatment cost which was defined as the sum of the hospitalization cost and time cost. The Human Capital Approach was used to evaluate time cost based on Overall Length of Stay (OLS) and Annual Net Income (ANI) per capita for rural residents in Henan province during hospitalization (18) (**Supplementary Table 1**). Expenses for a caregiver were taken into consideration and the time cost for each hospitalization was calculated from Eq. (2) as follows:


(2) $$Time\;Cost=OLS\times \frac{ANI}{365}\times 2$$

Costs were reported in the form of mean (upper quartile, lower quartile) to describe the average level and degree of dispersion since they are usually with skewed distributions (19). A discount rate of 3% was used to annualize capital investments to year 2018. Costs were adjusted by the Chinese consumer price index for medical goods and services (16, 20). Currency was converted from Chinese Yuan to U.S. dollars using purchasing power parity exchange rates of 2018 ($1=¥3.55) (21).

### Statistical Analysis

Characteristics of patients in the two arms of the “Perfect Cohort” were compared using the χ^2^ test and the rank-sum test for categorical and continuous variables respectively. Univariate logistic regression was applied to investigate impact factors of treatment costs. The temporal trends of accumulated total treatment costs for two arms stratified by cancer type were evaluated within one year after the first hospitalization. The average hospitalization cost per case was divided into nine pre-defined classifications in the NCMS system. Data management and statistical analysis were conducted using R version.3.5.1. All tests were two-sided and had a significance level of 0.05 unless otherwise specified.

All authors had access to the study data and all authors reviewed and approved the final manuscript.

## Results

### Study Subjects

**Figure 2:**
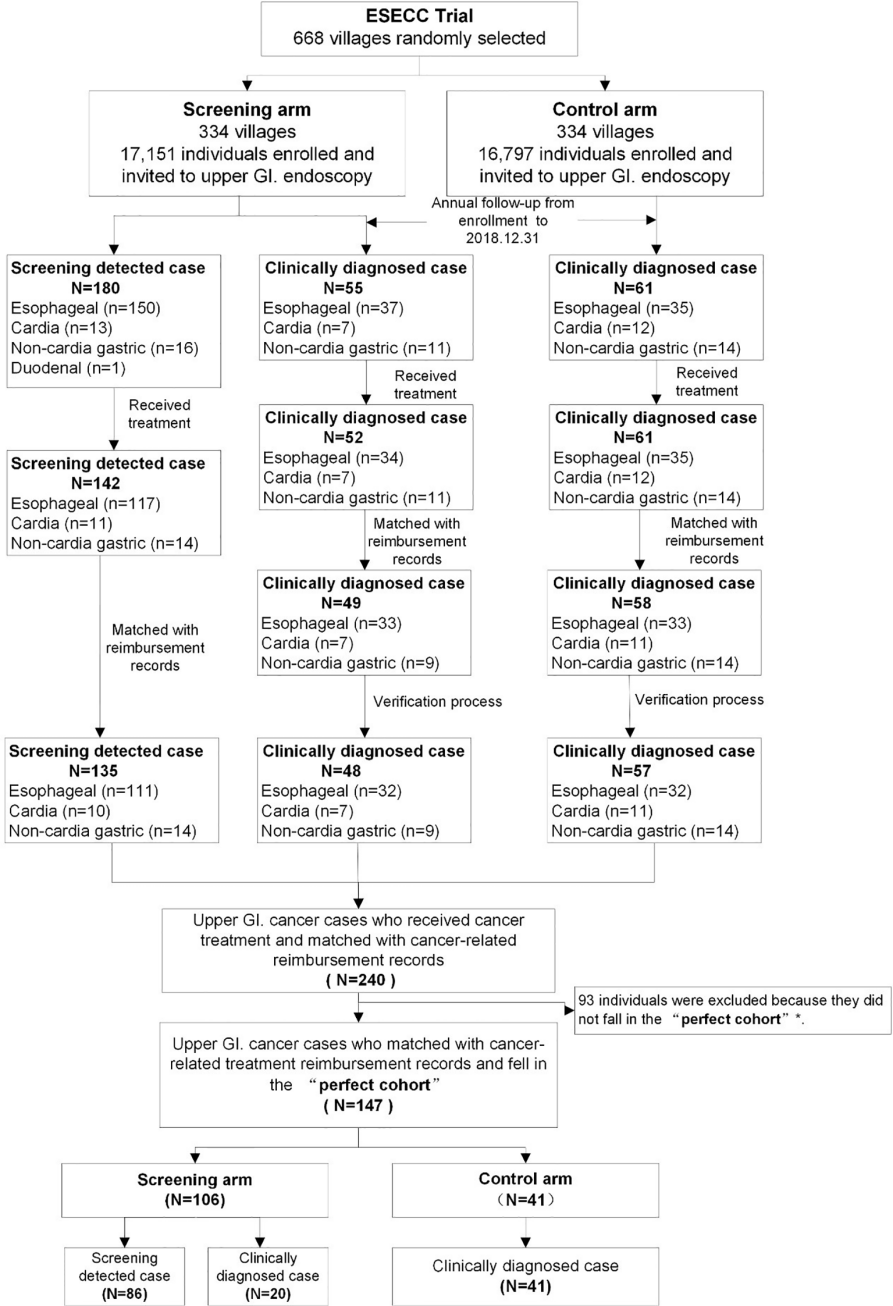
Flow of upper G.I. cancer cases included in the “Perfect Cohort” from the ESECC trial. *The “Perfect Cohort” refers to all upper G.I. cancer cases from both arms who were diagnosed within 857 days (the shortest follow-up time by the end of 31st December 2018 among all cases) after their enrollment in the ESECC trial.

A total of 180 cancers detected by screening (150 ECs,13 cardial cancers, 16 non-cardial gastric cancers and one duodenal cancer) and 55 clinically diagnosed (37 ECs, 7 cardial cancers, 11 non-cardial gastric cancers) cases of upper G.I. cancer were reported in the screening arm. Among these cancers 142 (117 ECs, 11 cardial cancers and 14 non-cardial gastric cancers) and 52 (34 ECs, 7 cardial cancers and 11 non-cardial gastric cancers) respectively received treatment. (**Figure 2**) In the control arm, 61 incident cancer cases (35 ECs,12 cardial cancers, and 14 non-cardial gastric cancers) were diagnosed and treated during the follow-up period. Detailed cost data were available for 94% (240/255) of these treated cancer cases.

In the uniform follow-up time period of 857 days following enrollment, a total of 147 of the 240 cases were included in the “Perfect Cohort”, including 41 clinically diagnosed cases in the control arm, and 106 cases (20 clinically diagnosed and 86 screening-detected) in the screening arm. The 147 cases under study and the 93 cases which were excluded were balanced in terms of age at enrollment (P=0.104), type of case (clinically diagnosed or screening-detected) (P=0.350), gender (P=0.890), education (P=0.999), occupation (P=0.999) and cancer site (P=0.608) (**Supplementary Table 2**). Eventually, with a “Standard observation window” of one year, a total of 344 hospitalization reimbursement records for these 147 patients were eligible for the following analysis.

### Comparison of Patient Characteristics

Socio-demographic characteristics including age at diagnosis, gender, occupation, and yearly household income were balanced in the two arms of the study. (**Table 1**) Regarding clinical features, a higher proportion of EC was detected in the screening arm (77% *vs.* 59%, P=0.023). Patients in the screening arm were generally earlier stage (67% were stage 0-I), while in contrast most patients (81%) in the control arm were stage III-IV. Accordingly, patients in the screening arm were more likely to receive early endoscopic treatment (12%) or radical resection without adjuvant treatment (62%) compared with the control arm (4% and 24%). Patients from the screening arm had fewer episodes of hospitalization (median 1 *vs.* 2) as well as a shorter accumulated median LOS per case (21 days *vs.* 39 days).

**Table 1:** Social-demographic and clinical features of subjects in the “Perfect Cohort” from the ESECC ^a^ trial.

	Control arm (N = 41)	Screening arm (N = 106)	*P* value
Age at diagnosis *median (quartile)*	64 (60; 67)	64 (62; 67)	0·419
Gender			
Male	28 (68%)	60 (57%)	0·260
Female	13 (32%)	46 (43%)	
Education level			
Middle school or above	15 (38%)	29 (28%)	0·317
Primary school or below	25 (62%)	73 (72%)	
Occupation			
Manual worker	39 (98%)	103 (99%)	0·480
Technical staff	1 (2%)	1 (1%)	
Household yearly income (USD) per capita *median (quartile)*	845 (282; 2,817)	563 (0; 1,972)	0·215
Number of cancer cases by site			
Esophageal cancer	24 (59%)	82 (77%)	0·023*
Cardial cancer	9 (22%)	7 (7%)	
Non-cardial gastric cancer	8 (19%)	17 (16%)	
Stage at diagnosis			
0-I	1 (5%)	58 (67%)	<0·001*
II	3 (14%)	21 (25%)	
III	8 (36%)	3 (3%)	
IV	10 (45%)	4 (5%)	
Therapy			
Endoscopic treatment	2 (4%)	13 (12%)	<0·001*
Single radical resection	10 (24%)	66 (62%)	
Radical surgery combined with radiotherapy or chemotherapy	13 (32%)	20 (19%)	
Radiotherapy or (and) chemotherapy	8 (20%)	4 (4%)	
Supportive care	8 (20%)	3 (3%)	
Frequency of hospitalization per case *median (quartile)*	2 (1; 4)	1 (1; 2)	<0·001*
Total length of stay (days) in hospital per case *median (quartile)*	39 (19; 64)	21 (16; 34)	0·011*

a ESECC (Endoscopic Screening for Esophageal Cancer in China) randomized controlled trial (Clinical trial: NCT01688908).

*Variables with P value < 0·05.

### Comparison of Economic Burden Between Arms

The economic burden stratified by cancer type was evaluated at both the individual level and the societal level (**Table 2**). Due to down staging and less costly treatment for cancer cases at earlier stages in the screening arm, average time cost per EC case ($573 vs. $993) and average hospitalization costs per EC case ($12,173 *vs.* $16,852) were lower than in the control arm. The out-of-pocket hospitalization cost after reimbursement ($5,972 *vs.* $7,557) was also lower. In spite of the lower individual economic burden in the screening arm, nearly four times the number of patients (80 *vs.* 21) compared with the control arm suffered catastrophic medical expenditures as gauged by the general standard wherein out-of-pocket expenses exceeded 40% of capacity to pay (15, 22). As a result, at the societal level, the overall treatment cost (sum of hospitalization cost and time cost) for all EC patients in the screening arm ($1,045,119) was 2.44 times that in the control arm ($428,292), as many cancer cases were identified in screening and were thus treated in advance (82 *vs.* 24).

**Table 2:** Treatment costs for subjects included in the “Perfect Cohort” from the ESECC ^a^ trial.

	Esophageal cancer		Cardial and non-cardial gastric cancer	
	Control arm (N = 24)	Screening arm (N = 82)	Control arm (N = 17)	Screening arm (N = 24)
Average time cost (USD) for hospitalization per case *mean (quartile)*	993 (397;1,461)	573 (312;614)	804 (373;1,256)	602 (394;889)
Average hospitalization cost (USD) per case *mean (quartile)*	16,852 (9,626;25,551)	12,173 (8,662;13,725)	19,876 (5,232;27,805)	15,784 (8,406;23,070)
Average treatment cost (USD) per case *mean (quartile)* ^b^	17,845 (10,148;27,145)	12,745 (9,023;14,434)	20,680 (5,573;29,197)	16,386 (8,796;24,006)
Average out-of-pocket hospitalization cost per case *mean (quartile)*	7,557 (4,012;10,786)	5,972 (4,231;6,639)	10,605 (2,248;14,586)	7,933 (4,212;12,120)
Number of cases of catastrophic health expenditure ^c^	21 (88%)	80 (98%)	15 (88%)	23 (96%)
Total treatment cost for all cancer patients	428,292	1,045,119	351,557	393,261

a ESECC (Endoscopic Screening for Esophageal Cancer in China) randomized controlled trial (Clinical trial: NCT01688908).

b Treatment cost in this study was calculated as the sum of hospitalization cost and time cost.

c Catastrophic health expenditure was defined by WHO as out-of-pocket costs exceeding 40% of patient capacity to pay, and Per Capita Disposable Income of rural residents in Hua County in 2017 was used as a substitutional index for capacity to pay in this study, amounting to 10906 RMB (3,072 USD).

Similar results were observed for cardial and non-cardial gastric cancer. Individual economic burden such as average time cost ($602 *vs.* $804), average hospitalization cost ($15,784 *vs.* $19.876) and average out-of-pocket hospitalization cost ($7,933 *vs.* $10,605) were lower in the screening arm, while in the screening arm more families suffered catastrophic health expenditures (23 *vs.* 15). Moreover, the overall treatment cost in the screening arm ($393,261) was 1.12 times that in the control arm ($351,557).

**Figure 3:**
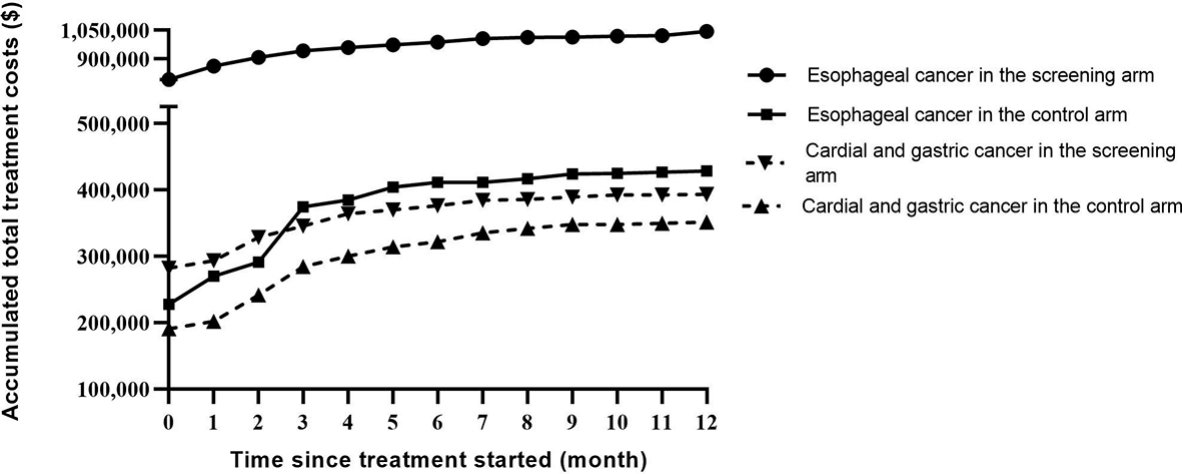
Temporal trends of accumulated treatment costs stratified by cancer type in the two study arms.

In temporal trend analysis, total treatment costs in both arms increased in an almost “parallel” manner within one year after the start of treatment, and cost in the screening arm remained higher than that in the control arm in both cancer types. The absolute difference in total treatment cost comparing the two arms of the study was much larger for EC than that for cardial and non-cardial gastric cancer (**Figure 3**)

Among the nine classifications, the greatest overspending for average hospitalization cost per case in the control arm resulted from radiation treatment fees ($2,716 *vs.* $1,299) and medication fees for chemotherapy ($6,518 *vs.* $4,547). In contrast, surgical fees for endoscopic treatment and radical resection were lower in the control arm ($724 *vs.* $1,039) (**Supplementary Figure 2**). This is consistent with the finding that a higher proportion of patients in the screening arm received early treatment such as endoscopic treatment or radical resection without adjuvant therapy which proved to be less costly than complicated therapies for advanced stage cancer (**Supplementary Table 3**).

## Discussion

In China, population level cancer screening programs have typically been funded and initiated by the national or local government, and have been implemented by local health care facilities. This approach is different from that in many western countries. In such pattern in which cancer screening was provided like a public service and welfare, during formulation of policy and budget planning most attention has been focused on the direct cost of screening, while incidental economic burden on cancer patients, households and target communities has been largely ignored. That is, resources for cancer screening are focused on screening intervention, and little attention is paid to the subsequent treatment phase. Cancer treatment costs resulting from screening have been borne mainly by patients and medical insurance sectors. Therefore, seen from a more comprehensive societal perspective, there is need for accurate evaluation of such economic burden in order to gain the attention of policymakers, and help anticipate potential financial impact on target communities. This will facilitate rational allocation of resources, allowing for compensation to patients and medical insurance departments when formulating budget plans. We thus made use of claims data from the health insurance system to estimate cancer-related treatment costs for upper G.I. cancer cases in the ESECC trial, and demonstrated the financial influence of screening. This research may shed light on this underappreciated problem in cancer screening programs worldwide, and provide insight for formulation of cancer screening policy in undeveloped regions.

In this study, individual economic burden of cancer treatment was relatively less in the screening arm, as a higher proportion of cancer cases were diagnosed in earlier stages, and therefore received less costly therapies such as endoscopic treatment and single radical resections. However, considering the low socio-economic level in targeted communities, the out-of-pocket treatment costs for most patients met the standard of catastrophic medical expenses, and the number of families suffering catastrophic health expenditure was much higher in the screening arm (103 *vs.* 36). We must keep in mind that such a significant influence on patient quality of life is worthwhile only when the effectiveness and cost-utility of cancer screening proves beneficial to participants, and this crucial evidence should be derived only from population-level randomized controlled trials.

The economic burden of cancer treatment is also stressful on the local community. In comparison with a “natural” population, many asymptomatic cancer cases would be identified in advance by screening. This is true in particular for cancer types of high-prevalence, which would lead to many more cases appearing in the screening arm (82 ECs) than in the control arm (24 ECs) within a time period where there are equal lengths of observation. Consequently, total demands for medical care and societal health expenditure increase soon after screening intervention. A sudden increase in health expenditure may cause significant problems throughout the medical system, especially when several screening programs are under way simultaneously which may magnify this effect. In this study, a “Perfect Cohort” and a “standard observation window” were constructed to eliminate all possible imbalances between the study arms. This allows a balanced comparison of cancer treatment costs, and we found that the total treatment cost for EC cases from the screening cohort ($1,045,119) was over twice that of its parallel control ($428,292). In contrast, for cardial and non-cardial gastric cancer with lower incidence risk in targeted communities, overall treatment costs for patients from the screening cohort ($393,261) was only 112% of that for the control arm ($351,557). This indicates that the higher the cancer risk is in a given population, the larger the financial impact for treatment cost introduced by cancer screening may be. Apart from the potential benefit of clinical down-staging and improved survival conferred by screening, more attention must be paid to the pressure which the medical insurance department may unexpectedly face due to a sharp increase in overall medical expenses caused by the great numbers of cancer cases detected in the screening. Therefore, the magnitude of unexpectedly explosive treatment costs should be assessed to facilitate appropriate compensation to the local health insurance department when planning cancer-screening budgets in areas of limited resources.

This study has three strengths. First, it has a high-quality control which serves as a baseline reference. Parallel and random control in a randomized controlled trial was used to achieve high comparability between groups. Second, there is parity of case inclusion and cost collection in the two study arms. Cancer treatment is often a prolonged undertaking with multiple hospitalization records, and case numbers and length of observation time have significant impact on total cost estimation. We constructed a “Perfect Cohort” and set a “Standard Observation Window” to abolish potential imbalances in cost estimation between study arms. Third, high-quality data is used to generate a precise estimation of economic burden. Our calculation of treatment cost was based on detailed first-hand medical claims data with accurate costs, excellent coverage of studied subjects and complete records of treatment in all health facilities involved.

This study has two limitations. First, this is a single-center study, and there may be variations in the severity of financial impact after screening due to different economic development levels, cancer type, disease burden, insurance coverage, response rate of screening and treatment cost in other communities. For example, a lower level of economic development and heavier disease burden are more likely to induce hardship due to the financial impact of cancer screening. Hence, the capacity for generalization of our conclusion requires broader evidence from other populations. Secondly, costs within the first year of treatment were adopted as an approximation of life-time costs which is the optimal choice for estimation of overall economic burden. However, considering the “L-shaped” trend of treatment cost flow over time which was identified in our study subjects, this will not impact our main conclusions.

## Conclusions

Population-level cancer screening in resource-limited areas will lead to further occurrences of catastrophic medical expenses among patients and sharply increase the total economic burden of cancer treatment on local government, especially for cancer types of high-prevalence. Greater financial support for the patients in this situation, as well as to health facilities and medical insurance departments in targeted communities should be taken into consideration when a comprehensive budget plan for cancer screening programs is under formulation.

## Data Availability Statement

Partially anonymized datasets from which identifying information has been removed will be made available upon request for purposes of academic research. Requests should be directed to YK and ZHe (Corresponding authors). Data will be available once all secondary papers which have been planned are published.

## Ethics Statement

The studies involving human participants were reviewed and approved by the Institutional Review Board of the Peking University School of Oncology, Beijing, China. The patients/participants provided their written informed consent to participate in this study.

## Authors Contributions

YK, ZHe, and FuL contributed to the conception and design of the study. YH, CG, LL, FeL, ML, ZL, YP, FaL, YL, ZHu, and HC contributed to the acquisition of data. FuL and ZHe contributed to data analysis. YK, ZHe, FuL contributed to interpretation of data and checking results. YK, ZHe, FuL contributed to drafting the manuscript, which was reviewed and approved by all coauthors.

## Funding

This work was supported by the National Science & Technology Fundamental Resources Investigation Program of China (No. 2019FY101102), the National Key R&D Program of China (No. 2021YFC2500405), the National Natural Science Foundation of China (No. 82073626), the Beijing-Tianjin-Hebei Basic Research Cooperation Project (No. J200016), the Digestive Medical Coordinated Development Center of Beijing Hospitals Authority (No. XXZ0204).

## Conflict of Interest

The authors declare that the research was conducted in the absence of any commercial or financial relationships that could be construed as a potential conflict of interest.

## Publisher’s Note

All claims expressed in this article are solely those of the authors and do not necessarily represent those of their affiliated organizations, or those of the publisher, the editors and the reviewers. Any product that may be evaluated in this article, or claim that may be made by its manufacturer, is not guaranteed or endorsed by the publisher.

## Abbreviations

EC, Esophageal Cancer; G.I., gastrointestinal.
