# Supplementary material for: Economic Burden Conferred by Population-Level Cancer Screening on Resource-Limited Communities: Lessons From the ESECC Trial
Source: Front Oncol. 2022 Mar 21;12:849368. doi: 10.3389/fonc.2022.849368 (PMC8977508; doi:10.3389/fonc.2022.849368)
Supplement: Supplementary file 1 [file Image_1.pdf]

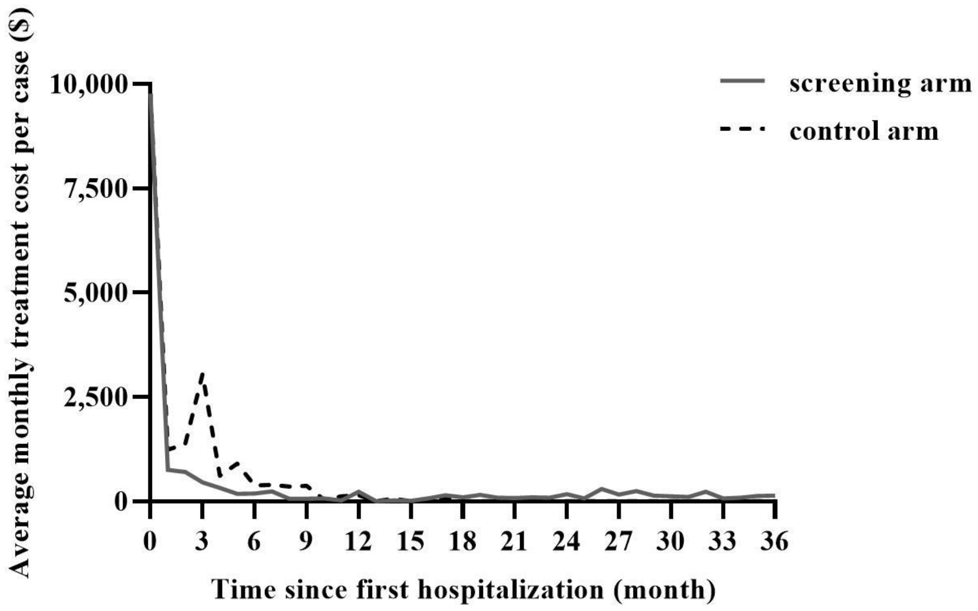

Supplementary Figure 1 Time trends for monthly average treatment cost per patient under observation since the first hospitalization in two arms of the ESECC trial
