# Supplementary material for: Economic Burden Conferred by Population-Level Cancer Screening on Resource-Limited Communities: Lessons From the ESECC Trial
Source: Front Oncol. 2022 Mar 21;12:849368. doi: 10.3389/fonc.2022.849368 (PMC8977508; doi:10.3389/fonc.2022.849368)
Supplement: Supplementary file 2 [file Image_2.pdf]

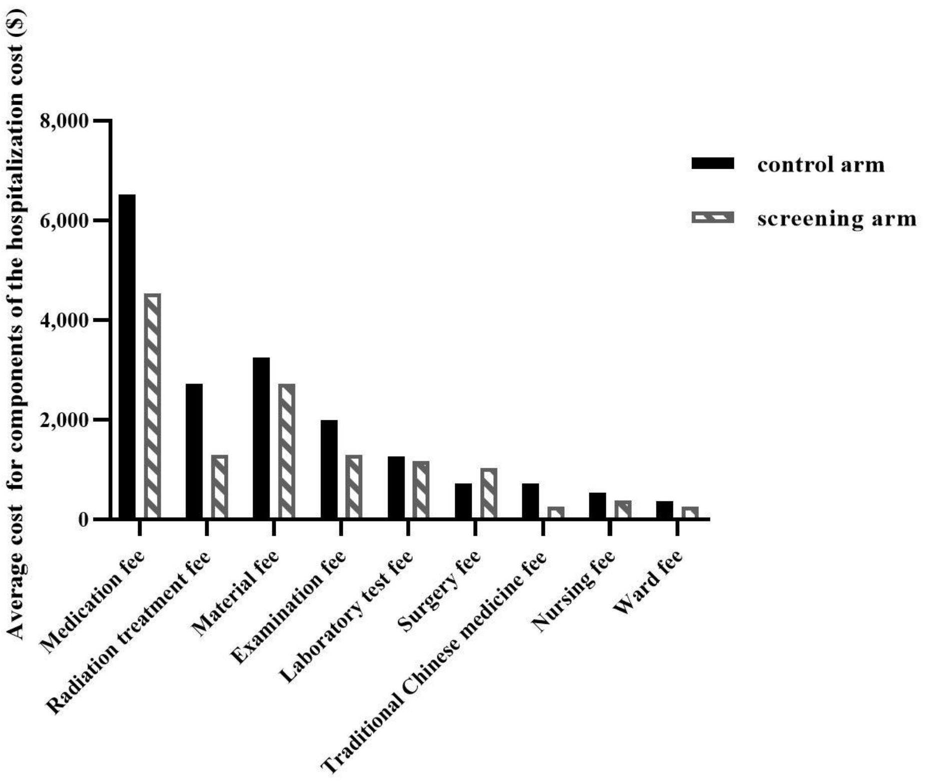

Supplementary Figure 2 Average cost for each component of the hospitalization cost per case in two arms of the ESECC trial.
