# Supplementary material for: Economic Burden Conferred by Population-Level Cancer Screening on Resource-Limited Communities: Lessons From the ESECC Trial
Source: Front Oncol. 2022 Mar 21;12:849368. doi: 10.3389/fonc.2022.849368 (PMC8977508; doi:10.3389/fonc.2022.849368)
Supplement: Supplementary file 3 [file Table_1.pdf]

Supplementary Table 1. Basic socio-economic statistics adopted in cost calculations.

Year	Annual Net Income (ANI) per capita of rural residents in Henan province (RMB)	Chinese Consumer Price Index for medical goods and services
2012	7524.9	101.7
2013	8475.3	101.5
2014	9416.1	101.7
2015	10852.9	102.7
2016	11696.74	103.8
2017	12719.18	106
2018	12719.18	105.5
