# Supplementary material for: Economic Burden Conferred by Population-Level Cancer Screening on Resource-Limited Communities: Lessons From the ESECC Trial
Source: Front Oncol. 2022 Mar 21;12:849368. doi: 10.3389/fonc.2022.849368 (PMC8977508; doi:10.3389/fonc.2022.849368)
Supplement: Supplementary file 4 [file Table_2.pdf]

Supplementary Table 2. Selection bias analysis between the 93 excluded cases and the 147 cases remaining in the "Perfect cohort".

**Variable list**	**Perfect Cohort**	**93 excluded cancer cases**	***P* value**
Number of cases (N)	147	93	
Age at diagnosis median (quartile)	64(61; 67)	67(64; 69)	<0.0001*
Age at enrollment median (quartile)	64(61; 67)	63(60; 66)	0.10
Case resources			
Screened cases	86(59%)	48(52%)	0.35
Clinical cases	61(41%)	45(48%)	
Case groups			
Screening arm	106(72%)	77(83%)	0.060
Control arm	41(28%)	16(17%)	
Gender			
Male	88(60%)	57(61%)	0.89
Female	59(40%)	36(39%)	
Education level			
Middle school or above	44(31%)	27(30%)	0.99
Primary school or below	98(69%)	62(70%)	
Occupation			
Manual worker	142(99%)	86(99%)	0.99
Technical staff	2(1%)	1(1%)	
Household yearly income (USD) per capita median (quartile)	845(0; 2,183)	1126(0; 2,817)	0.42
Number of cancer cases by site			
Esophageal cancer	106(72%)	68(73%)	0.61
Cardia cancer	16(11%)	13(14%)	
Non-cardia gastric cancer	25(17%)	12(13%)	
Stage at diagnosis			
0	25(23%)	19(36%)	0.080
I	34(32%)	10(19%)	
II	24(22%)	6(11%)	
III	11(10%)	9(17%)	
IV	14(13%)	9(17%)	

*Variables with P value <0.05.
