# Supplementary material for: Economic Burden Conferred by Population-Level Cancer Screening on Resource-Limited Communities: Lessons From the ESECC Trial
Source: Front Oncol. 2022 Mar 21;12:849368. doi: 10.3389/fonc.2022.849368 (PMC8977508; doi:10.3389/fonc.2022.849368)
Supplement: Supplementary file 5 [file Table_3.pdf]

Supplementary Table 3 Univariate analysis stratified by arms on impact factors associated with treatment costs for upper GI. Cancer cases in the "perfect cohort" from the ESECC trial (2018 USD)

**Variable list**	**Average treatment cost (quartile)**			**Average treatment cost (quartile)**		
	**N**	**Control arm (N=41)**	**P value**	**N**	**Screening arm (N=106)**	***P* value**
Gender						
Male	28	21,405(10,766; 32,787)	0.14	60	14,638(9,226; 17,548)	0.11
Female	13	13,884(3,245; 23,280)		46	12,176(8,508; 14,171)	
Age at diagnosis						
＜65	22	22,441(6,393; 32,943)	0.29	40	12,805(8,995; 15,119)	0.79
≥65	19	15,060(9,312; 17,139)		66	14,033(8,463; 17,668)	
Hospital grade						
Private hospital	1	763	0.026*	0	-	0.0030*
Secondary degree public hospital	14	12,638(4,533; 15,384)		39	10,919(8,526; 12,841)	
Tertiary public hospital	26	23,160(12,051; 33,029)		67	15,112(9,689; 19,561)	
Treatment cost by cancer site						
Esophageal cancer	24	17,845(10,148; 27,145)	0.82	82	12,745(9,023; 14,434)	0.19
Cardia cancer	9	24,233(5,573; 33,031)		7	13,303(10,718; 15,775)	
Non-cardia gastric cancer	8	16,682(6,928; 25,003)		17	17,655(8,255; 25,957)	
Stage at diagnosis						
0-I	1	10,579	0.17	58	11,059(8,917; 13,260)	0.0060*
II	3	19,878(12,467; 23,989)		21	15,069(10,259; 16,852)	
III	8	29,599(21,987; 29,369)		3	26,877(19,691; 33,275)	
IV	10	16,098(6,393; 22,864)		4	20,200(15,622; 23,844)	
Therapy						
Endoscopic treatment	2	9,548(6,564; 12,532)	<0.0001*	13	5,991(3,140; 7,269)	<0.0001*

Single radical resection	10	17,783(11,467; 25,837)	66	12,178(9,278; 13,369)
Radical resection combined with radiotherapy or chemotherapy	13	31,809(23,280; 37,822)	20	22,433(15,268; 25,967)
Radiotherapy or (and) chemotherapy	8	17,626(8,537; 24,387)	4	17,582(8,480; 23,591)
Supportive care	8	3,550(1,229; 3,313)	3	12,585(9,515; 18,325)

*Variables with P value <0.05.
